# Investigating the importance of self-theories of intelligence and musicality for students' academic and musical achievement

**DOI:** 10.3389/fpsyg.2015.01702

**Published:** 2015-11-05

**Authors:** Daniel Müllensiefen, Peter Harrison, Francesco Caprini, Amy Fancourt

**Affiliations:** ^1^Department of Psychology, Goldsmiths, University of LondonLondon, UK; ^2^Head of Psychology, Brain Can Do, Queen Anne's SchoolReading, UK

**Keywords:** musical ability, academic performance, self-concepts, personality, theory of intelligence, theory of musicality

## Abstract

Musical abilities and active engagement with music have been shown to be positively associated with many cognitive abilities as well as social skills and academic performance in secondary school students. While there is evidence from intervention studies that musical training can be a cause of these positive relationships, recent findings in the literature have suggested that other factors, such as genetics, family background or personality traits, might also be contributing factors. In addition, there is mounting evidence that self-concepts and beliefs can affect academic performance independently of intellectual ability. Students who believe that intelligence is malleable are more likely to attribute poor academic performances to effort rather than ability, and are more likely to take remedial action to improve their performance. However, it is currently not known whether student's beliefs about the nature of musical talent also influence the development of musical abilities in a similar fashion. Therefore, this study introduces a short self-report measure termed “Musical Self-Theories and Goals,” closely modeled on validated measures for self-theories in academic scenarios. Using this measure the study investigates whether musical self-theories are related to students' musical development as indexed by their concurrent musical activities and their performance on a battery of listening tests. We use data from a cross-sectional sample of 313 secondary school students to construct a network model describing the relationships between self-theories and academic as well as musical outcome measures, while also assessing potential effects of intelligence and the Big Five personality dimensions. Results from the network model indicate that self-theories of intelligence and musicality are closely related. In addition, both kinds of self-theories are connected to the students' academic achievement through the personality dimension conscientiousness and academic effort. Finally, applying the do-calculus method to the network model we estimate that the size of the assumed causal effects between musical self-theories and academic achievement lie between 0.07 and 0.15 standard deviations.

## Introduction

Musical abilities and engagement with music have been shown to be positively related to cognitive abilities (e.g., Chan et al., 1998; Ho et al., 2003; Schellenberg, 2006, 2011; Ruthsatz et al., 2008; Degé and Schwarzer, 2011) as well as pro-social behavior (Bastian et al., 2000; Harland et al., 2000; Broh, 2002; Kirschner and Tomasello, 2010) and academic performance (Weber et al., 1993; Gardiner et al., 1996) in secondary school students. However, much of the evidence for the positive effects of musical training on cognitive abilities comes from correlational studies with cross-sectional designs, where associations between musical variables and cognitive, social, or academic measures are observed with “snapshot” data collections, typically across several year groups of one or more schools. From cross-sectional data of this kind, it is generally difficult to infer whether musical abilities and musical engagement are the cause or the effect of enhanced cognitive and social abilities as well as academic performance (Schellenberg, 2011).

Nonetheless, evidence from a small number of existing interventional studies—where one group of students given special musical training is compared to a control group without musical training—seems to suggest that training on a musical instrument can indeed increase intelligence scores (Schellenberg, 2004) as well as academic grades (Ho et al., 2003). However, the effects found in these interventional studies (Schellenberg, 2004; Moreno et al., 2011) are usually smaller (Costa-Giomi, 1999; Moreno et al., 2009) compared to the effects found in correlational studies (Schellenberg, 2006, 2011) or even non-existent (Mehr et al., 2013).

One suspected reason for the larger effect sizes generally found in correlational studies is the presence of confounding variables that potentially affect cognitive, social, and academic outcome measures, as well as musical engagement and abilities. Among the potentially confounding variables investigated in the past are the socio-economic status (SES) of the parents' home (Corrigall et al., 2013), genetic endowment (Mosing et al., 2014a), personality factors (Corrigall et al., 2013), and beliefs and concepts about one's own abilities (Degé et al., 2014). While these confounding variables can be controlled for in studies with randomized controlled designs, thereby assessing the unbiased effect of music interventions on outcomes in other domains, it is also interesting to record these confounding variables in observational designs; sometimes, a confounding variable may in fact turn out to be an important mediator of an experimental effect. A similar approach to account for the influence of confounding variables has been recently demonstrated in a study by Hille and Schupp (2015) using a large sample taken from a representative socio-economic panel. They show how it is possible to control for a large number of potentially confounding variables in a cross-sectional design with the help of propensity score matching (Stuart, 2010). After controlling for confounding variables, Hille and Schupp (2015) found significant positive effects of musical training (i.e., music lessons on an instrument for a sustained period of time) on school grades and personal academic ambition in their sample of adolescents.

The current study represents the starting point of a longitudinal study that will take repeated measurements of cognitive, social, and musical abilities, as well as academic performance, on the same secondary school children over 5 years. The main question of this longitudinal study concerns the *co-development* of musical competencies, intelligence, social skills, self-concepts, and beliefs. Specifically, we want to ask whether increased engagement with music that sets in for some children during adolescence, has positive effects on intelligence and social skills. We also want to consider whether such transfer effects are posterior to increased musical engagement or actually precede it.

A special focus of the longitudinal study is the question of how adolescent's beliefs and motivations affect their academic and musical development. Over the course of adolescence, children continually gain more autonomy concerning their studies, both academic and musical, and the ability to motivate oneself to engage with homework or practice a musical instrument without the direct supervision of an adult becomes increasingly important. This study aims to investigate what causes one adolescent to engage with academic or musical study and another to lose motivation.

One important contributing factor to children's motivation is how they respond to failure. The effect of failure on motivation can vary substantially from child to child (Diener and Dweck, 1980). Some adolescents attribute their failures to a lack of ability, and therefore view the difficulty as insurmountable. Their opinion of their own intelligence drops, and in order to avoid further failure they stop investing effort in the task. Diener and Dweck (1980) termed this reaction the *helpless* response pattern; similar response patterns are identified in Seligman's (1975) writings on *helplessness* and expanded upon in Weiner's (1986) *attributional theory of motivation*. In contrast, some adolescents exhibit a *mastery-oriented* pattern (Diener and Dweck, 1980) where they see failure as challenge to be overcome by hard work. This latter pattern is beneficial to the student: Mastery-oriented students express greater subject interest (Cury et al., 1996), invest more effort (Dupeyrat and Mariné, 2005), and perform better academically (Dupeyrat and Mariné, 2005), especially when faced with challenging material (Licht and Dweck, 1984). The causes of these different behavior patterns in responses to failure are assumed to be rooted in the students' *theories of intelligence* (Hong et al., 1999). Students who believe that intelligence is malleable (*incremental theorists*) are more likely to attribute poor performances to effort rather than ability, and are more likely to take remedial action to improve their performance as a result (Hong et al., 1999). They enter academic situations with *learning goals*, meaning that they prioritize their own intellectual development over how intelligent they appear to others (Elliott and Dweck, 1988; Robins and Pals, 2002). Incremental theorists therefore tend to be mastery-oriented. In contrast, students who believe that intelligence is fixed (*entity theorists*) are more likely to attribute poor performances to a lack of ability, and are less likely to respond to poor performance by increasing effort (Hong et al., 1999). They enter academic situations with *performance goals*, prioritizing positive assessments over learning and avoiding challenging situations where their ability might be tested (Elliott and Dweck, 1988; Robins and Pals, 2002). When they do meet with failure, it has a strong negative effect on their academic self-concept. Possessing an entity theory of intelligence therefore leads to helpless behavior.

Despite theories of intelligence being uncorrelated with general cognitive ability (Dweck et al., 1995; c.f. Robins and Pals, 2002), theories of intelligence do reliably predict academic performance in students, especially when the learning environment is a challenging one. This relationship has been illustrated by a number of longitudinal studies (Henderson and Dweck, 1990; Dweck and Sorich, 1999; Robins and Pals, 2002) and the relationships between students' academic development, their motivational patterns, and their theories of intelligence are therefore well understood. Less explored, however, is the way in which these relationships might extend to musical development. Learning to play a musical instrument is a challenging task that relies heavily on the student's autonomous motivation, since the majority of instrumental practice tends to occur unsupervised. It seems likely then that students who respond well to failure ought to progress better in music, and indeed there is some preliminary evidence to support this hypothesis: O'Neill (1996 cited in O'Neill, 1997) found that children who exhibited mastery-oriented behavior on a problem-solving task displayed significantly higher performance achievement after a year of formal instrumental tuition than helpless students taking the same tuition. Mastery-oriented behavior may therefore be particularly important for successful musical development. As yet unexamined, however, is the question of what factors cause a student to take a mastery-oriented attitude toward music. It has been shown that academic mastery-oriented behavior is fostered by an incremental theory of intelligence and it may be that in an analogous fashion, musical mastery-oriented behavior may be fostered by an incremental theory of musical ability. According to this hypothesis, students who believe that musical ability or talent is learned, rather than innate, will direct their musical engagement toward learning goals rather than performance goals, resulting in an adaptive mastery-oriented approach to music, and improved long-term musical achievement.

Thus, we aim to investigate in the present study to what degree beliefs about the nature of intelligence and about the nature of musical ability are related to each other and how both of these beliefs might be connected to academic performance. In addition, we ask how musical self-theories are related to current and past active musical behaviors including musical training and how this in turn might affect a range of musical listening abilities and skills. If it turns out that self-theories of intelligence and musical abilities are indeed connected to academic effort, academic achievement, musical training, and listening skills, then further questions can be asked to which other factors these self-theories are associated, such as personality traits which are considered rather stable traits over time.

Because the study reported in this paper is a “level-0” cross-sectional precursor to a series of yearly waves of longitudinal measurements over the following years, we cannot report any developmental trends derived from the data of the same participants yet. However, the analysis of the cross-sectional data of this study can provide baseline scores for all cognitive and perceptual tests and self-reported variables, differentiated by age. Secondly, the data from this cross-sectional study serve to investigate correlational links between the core factors of interest: personality, intelligence, self-theories and self-concepts, musical activity, academic performance and musical listening skills. In the previously discussed literature bivariate correlational links have been reported between almost all of these factors, but it is unclear which of these factors are still significantly correlated with each other when all other factors are taken into account. Thus, we aim to construct a network of conditional independence relationships including all of the core factors of interest using modern methods of graphical modeling in multivariate datasets (e.g., Spirtes et al., 2000; Pearl, 2009). Finally, we aim to investigate specifically the effect of self-theories (i.e., “Academic Self-Theories and Goals” and “Musical Self-Theories and Goals”) on academic performance and musical listening skills within this network of conditional independence relationships. This final analysis starts from the assumption that there is a causal link from self-beliefs to academic and musical performance. The analysis aims to answer the question of how strong an effect on academic performance would be achieved if the beliefs of an individual would be moved from an entity theory of intelligence and musicality to an incremental theory of intelligence and musicality. Given that academic and musical self-theories are presumed to be partly malleable, the answer to this question can provide an indication of whether changing the belief systems of adolescents through an intervention could potentially affect academic and musical efforts and ultimately academic and musical achievements. To be clear, a definite answer as to whether such an intervention could generate positive effects can only be expected from a randomized controlled experiment. However, using Pearl's do-calculus (Pearl, 2009) we can ask from the present data how strong this effect would be, if the assumed causal relationship would indeed exist.

In sum, this study aims to determine the interplay of self-theories, personality traits, musical activities and musical abilities, and educational achievement in form of a network model. Specifically, we ask to which other factor musical and academic self-theories are related and whether and how are they connected to academic achievement. Finally, if a causal link between self-theories and academic achievement exists, we aim to derive an estimate for the effect size of this causal connection, which can serve as a hypothesis for subsequent research using an interventional design.

## Methods and materials

The study used a correlational design where participants had to complete a test battery consisting of five different perceptual/cognitive tasks and eight self-report measures. In the following we provide a short description of each test. For published tests and questionnaires from the existing literature we keep the description as short as possible and will point to the corresponding references for their psychometric properties. For the novel tests that were designed for use in this study we provide the relevant details on test construction and psychometric benchmark figures. In addition to the tests described below the students also responded to a version of the Crandall Social Desirability Scale for Children (CSDSC, Carifio, 1994) in order to be able to correct for potential effects of social desirability and the five-factor listening test of musical preferences (Rentfrow et al., 2011) to gather baseline data for the planned longitudinal study on the co-development of musical abilities, preferences, personality and cognitive abilities. But due to space restrictions results from these tests are not reported in this paper.

Overall, we aimed for efficiency when selecting and constructing the tests used in this study. For the perceptual/cognitive tests this led to the adoption of computerized adaptive tests (CATs; e.g., Latu and Chapman, 2002) based on rigorous item response models (e.g., de Ayala, 2009). In CATs each subsequent test item is chosen to match optimally the estimated ability level of the participant's ability level, based on the responses to all previous items. This means that tests can be considerably shorter (i.e., containing fewer items), while maintaining a high precision for the ability estimates (i.e., small confidence intervals) compared to tests that use the same items for all participants. Shortening the test length of some of our perceptual tests using CAT permitted us to administer a wide range of different tests while minimizing participant fatigue.

### Participants

Three hundred and twelve students from Queen Anne's girls school Caversham, UK, participated in the study. Participation was voluntary and consent was sought from the students' parents in advance. The study was approved by the departmental ethics committee at Goldsmiths, University of London. All participants were female and recruited from all year groups of the school and ages ranged from 10 to 18. The mean age was 14.14 years (*SD* = 1.92 years).

### Materials and procedure

Testing took place during normal school hours and participants were tested in groups in the school's computer labs where each participant was seated in front of a computer with headphones (Behringer HPM1000) attached to each machine. At the beginning of each testing session participants were presented with a short audio piece and instructed to adjust the volume to a comfortable level via the computer screen. All tests were administered via an online interface in two blocks separated by a short break. Participants were then instructed to work at their own pace through the series of tests. Participants took between c. 55 and c. 75 min in total to complete the two testing blocks.

#### Cognitive tests

##### Intelligence

We assessed general intelligence using the MyIQ test (Chan and Kosinski, 2015), a computerized adaptive matrix reasoning test modeled on the Raven's Progressive Matrices. The test was developed by the Cambridge Psychometrics Centre, as part of the International Cognitive Ability Resource (ICAR) project, and items had previously been validated against the copyrighted version of the Raven's matrices. Each item contained a matrix with nine spatial slots, eight of which contained simple visual patterns, and the participant's task was to select a missing pattern to fit the ninth slot from eight potential choices provided. Each item was timed to a maximum of 2 min, after which the target matrix disappeared and participants had to make a choice to progress to the next item. A total of eight items was administered to participants using an adaptive procedure, with participant ability being estimated by Bayes modal estimation (Birnbaum, 1969) and each subsequent item being chosen to maximize the item information function for that ability level (Baker, 1992).

#### Musical tests

##### Beat perception

The Beat Perception Test aims to assess the participant's ability to process the beat in musical extracts. The test used in this study is related to the Beat Alignment Test developed by Iversen and Patel (2008) and the derived fixed-length Beat Perception Test of the Gold-MSI battery (Müllensiefen et al., 2014). In order to maximize ecological validity, the Beat Perception test used excerpts of real music from a variety of musical genres, obtained from an online music library (http://audionetwork.com), with each extract being trimmed to a length of approximately 5 s. The rational for drawing the musical extracts from a wide variety of genres is to avoid confounding effects of any particular musical genres that a subgroup of participants might be highly familiar or unfamiliar with. For each track the locations of the musical beats were identified by averaging across several performances of two expert drummers tapping in time to the excerpts. The test uses a two-alternative forced-choice (2AFC) task. In each trial, participants are presented with two renditions of the same musical track, both overlaid with a metronome-like “beep track.” In one version, the target, the beep track is exactly in time with the musical beat locations, while in the other version the beep track is displaced from the musical beat locations by a constant proportion of a beat. The participant's task is to identify which extract has the beep track on the beat. This requires the perception of the musical beat as a latent perceptual construct arising from a complex audio source as well as the comparison of this latent construct to the manifest beep track. At the time of the present study, the Beat Perception Test had been calibrated over an extended piloting period using data from 427 participants; on the basis of this data, we produced a 1-parameter IRT model with guessing parameter fixed at 0.5 (i.e., chance level in the 2AFC task). We then tested whether the model met the rigorous assumptions of IRT modeling (see e.g., de Ayala, 2009; DeMars, 2010): model fit (the data can be fit well by an IRT model), unidimensionality (all items measure the same trait), and local item independence (the response to one item does not depend on the response to another item after accounting for the trait being measured). The model showed an overall satisfactory fit to the data (*M*2 = 383.02, *df* = 324, *p* = 0.01, *RMSEA* = 0.03, *SRMSR* = 0.07), passed the test for unidimensionality (modified parallel analysis with 100 Monte Carlo samples, *p* = 0.44; Drasgow and Lissak, 1983), and local item independence as indexed by the χ^2^ statistic (none of the items having a χ^2^ value above the 0.05 significance level after Bonferroni correction, Chen and Thissen, 1997). Since the item difficulties were predicted very well by a measure of the displacement of the beep-track from the musical beat (Pearson's *r* = 0.93), we constructed a binomial logistic mixed effects regression model analogous to the 1-parameter IRT model, but using beep-track displacement as a continuous predictor. This model then permits the estimation of psychometric item difficulty of new items on the basis of their beep-track alignment. This therefore enables us to calibrate the difficulty of a large set of new stimuli belonging to a fine grid of difficulty levels for the CAT procedure.

The selection of stimuli in the adaptive procedure was constrained so that a participant never heard the same musical track more than once. Participants were introduced to the task by a training phase comprising detailed instructions, audio examples, and practice questions with feedback. The training phase was followed by the test phase, which comprised 25 trials. For each trial, both versions of the audio excerpt were played automatically separated by a 1 s silent gap. Participants were prompted to indicate whether the beep track was on the beat in the first or the second version of the audio excerpt. Participant abilities were estimated using Bayes modal estimation (Birnbaum, 1969), which returns a *z*-score typically within the bounds of −3 to 3.

##### Melodic memory

The Melodic Memory Test assesses the participant's short-term memory for melodies and is modeled on long-established cognitive paradigms from the literature (e.g., Bartlett and Dowling, 1980; Cuddy and Lyons, 1981). More specifically, it is designed as an “odd-one-out” variant of the melodic memory test that has been part of the Goldsmiths Musical sophistication Index (Gold-MSI, Müllensiefen et al., 2014). The current version of the test is a fixed-length version where participants respond to 20 items in total. For each item, the participant is presented with three versions of the same short melody, each starting a semitone higher but maintaining the same interval structure, except for one altered note in one of the three versions, called the “odd one out.” This “odd one out” version can occur in any one of three positions; the participant's task is to identify the position of the “odd one out” by clicking one of the response buttons “1,” “2,” or “3.” The difficulty of this task is varied systematically by changing the length of the melody and the nature of the melodic alteration. This version of the Melodic Memory Test was a non-adaptive prototype, designed to collect data for calibrating a future adaptive version while providing a reliable score calculated from the set of 20 items which were of equal difficulty for each participant. The base melodies for each item were produced algorithmically by the computational model Racchman-Oct2010 (Random Constrained Chain of Markovian Nodes; Collins et al., 2015), which takes as input a corpus of source music (monophonic or polyphonic) in a particular musical style, calculates a matrix of transition probabilities between every musical event and uses this transition matrix to generate new musical extracts in the style of the source corpus (for a more detailed account see Collins et al., 2015). It is possible to constrain the generation process according to various music-mathematical criteria. One constraint applied in this study was that no more than two consecutive events should come from the same melody, reducing the probability that the algorithm would replicate a segment of a source melody note for note. In the present study, the source corpus was the collection of Irish folk melodies from the Essen collection (Schaffrath, 1995) in simple triple time. All melodies produced by Racchman-Oct2010 were subsequently filtered to ensure that they contained no out-of-key notes, and transposed to the key of D major. For manipulating item difficulty we varied item length based on evidence showing that longer melodies are harder to encode (Brittin, 2000; Akiva-Kabiri et al., 2009). Five different levels of melody length were therefore employed, staggered in logarithmic steps (lengths of 6, 7, 9, 12, 16 notes). In addition we manipulated melodic contour (Dowling and Fujitani, 1971) and tonal key membership (Trainor and Trehub, 1992, 1994) to control item difficulty. The manipulated note in the odd melody version could either preserve melodic contour or it could violate it. We only used extreme contour violations, defined as manipulations where a “step up, step down” pattern was replaced with a “step down, step up” pattern, or vice versa. Based on a large corpus of evidence (see e.g., Dowling, 1978; Bartlett and Dowling, 1980; Massaro et al., 1980; Dowling et al., 1995; Cutietta and Booth, 1996) we hypothesized that item difficulty would be lowest when the melodic contours differed, as opposed to when only melodic interval and not contour differed. As a third factor contributing to item difficulty we manipulated the tonal implications of the altered note: The tonality of the melody could either be violated, by introducing a note outside the diatonic scale, or maintained, by keeping to the diatonic scale throughout. Since the original melodies were constrained to contain only in-key notes, changing a note to an out-of-key note is very likely to interfere with the tonal representation of the melody, resulting in a low item difficulty (i.e., making the “odd one out” easy to identify). Twenty melodies were generated by Racchman-Oct2010 for each level of melody length, producing 100 base melodies in total. These 100 melodies were crossed with the dichotomous variables “tonal implication” and “contour violation” to form 400 pairs of “odd one out” melodies and lures. Since, for any given melody length, item difficulty is presumed to be independent of the particular melody chosen, the five levels of melody length, two levels of contour difference, and two levels of tonality difference therefore combine factorially to produce 20 different classes of item difficulty. These pairs of “odd one out” melodies and lures were then used to produce triplets of melodies (two lures, one “odd one out”) for the experimental trials; since for any pair the “odd one out” can come first, second, or third, there were 400 × 3 = 1200 stimuli generated in total. Participants were introduced to the task with one audio example and two practice trials (one easy, one difficult item) where feedback was provided. Participants were free to repeat the example and practice trials if they felt that the task procedure was not clear. Subsequently, each participant was presented with 20 trials where each of the 20 item difficulty levels occurred exactly once. In each experimental trial, the three melodies were separated each by 1 s of silence. A 1-parameter IRT model with guessing parameter at 1/3 met the IRT test assumptions of good model fit (*M*2 = 183.54, *df* = 189, *p* = 0.60, *RMSEA* < 0.01, *SRMSR* = 0.05), unidimensionality (modified parallel analysis with 100 Monte Carlo samples, *p* = 0.44; Drasgow and Lissak, 1983), and local item independence as indexed by the χ^2^ statistic (none of the items having a χ^2^ value above the 0.05 significance level after Bonferroni correction, Chen and Thissen, 1997).

##### Sound similarity

The sound similarity test assesses the ability to make comparative judgments about very short excerpts from music recordings. The test is inspired by recent studies showing that ordinary listeners are able to make musical judgments from sound information alone. Gjerdingen and Perrott (2008) and Mace et al. (2011) showed that listeners are able to identify the genre of short musical clips from excerpts as short as 250 ms, while Krumhansl (2010) demonstrated that listeners familiar with the pop music genre were able to name the artist and song title after listening to similarly short clips. The test uses a sorting paradigm similar to the one used by Gingras et al. (2011) and Giordano et al. (2010). The test is described in Musil et al. (2013) and uses sixteen 800 ms excerpts from prototypical but lesser known songs from four different genres (rock, jazz, pop, hip hop) containing the song's full instrumentation but excluding any vocals. The participant's task was to listen to these short excerpts (as often as required) and to sort them into four groups of four items each by their similarity in sound. The term “genre” was deliberately avoided in the instructions and the participants were not instructed to focus on any specific sound features. Participants were allowed to change their sorting as often as necessary and there was no time constraint for the task. Due to the combinatorial nature of the scoring procedure item responses cannot be considered independent of each other, and therefore the reliability coefficient Cronbach's alpha is not appropriate. However, computing average split-half reliability of all 1296 splits on an earlier and more difficult version of the test using sortings of sixteen 400 ms excerpts by 137,339 participants (Müllensiefen et al., 2014) produced a reliability value of 0.71. The sound similarity test is scored using the corrected Rand index (Rand, 1971) which reflects the number of pairs of sound clips from the same genre that a participant placed into the same group. The scale of the Rand index ranges between 0 (chance level) and 1 (a perfect sorting solution).

#### Self-report measures

##### Personality

Personality was assessed using an extended version of the Ten Item Personality Inventory (TIPI, Gosling et al., 2003). The original test comprises 10 items, 2 for each personality trait, each one consisting of two attributes. Subjects are asked to report how much they identify with the attribute. Feedback from an initial pilot using the TIPI with children of the same age group made it clear that young children might not be familiar with all attributes in the test. Therefore, we added two synonyms to each item. The new attributes were selected from the lists of attributes associated with each personality facet of the Big Five Personality model reported in Goldberg (1990). The selection was done by a group of 11 independent judges, who were asked to select the most suitable attributes for the use in a personality inventory with adolescents. The resulting extended TIPI items can be found in the Supplementary Materials.

##### Musical sophistication

The Goldsmiths Musical Sophistication Index (Gold-MSI, Müllensiefen et al., 2014) was included in the battery in order to assess the participants' self-reported expertise and musical background. The Gold-MSI is a 39-item self-report scale that comprises five subscales (Active Musical Engagement, Perceptual Abilities, Musical Training, Singing Abilities, Emotional Engagement with Music) and one general factor (General Musical Sophistication). Initially developed for use with adults, the factor structure and internal reliability of the Gold-MSI have been replicated for its German translation (Schaal et al., 2014) and validated for the use with secondary school pupils in a large German sample of 11–19 year olds (Fiedler and Müllensiefen, 2015).

##### Concurrent musical activities

Because the self-report subscales of the Gold-MSI cover a broad period of an individual's life, including any past phases of musical activity, it was necessary to complement the Gold-MSI subscales with an additional measurement instrument that captures the degree to which school pupils are involved in musical activities at present. Secondary school education is a time when many adolescents come into contact with new educational and leisure time activities (such as music, sports, drama, visual arts, gaming, computer programming, etc.) and are able to test and develop their interests. For the purpose of the planned longitudinal study it is important to capture short-term boosts in musical activity that might be linked to opportunities at school, at home, or with one's peers. As a first step toward the development of a short self-report scale capturing concurrent musical activities, we collected qualitative information about the range of musical activities of secondary school students in a small pilot using 13 adolescents from across all year groups from Queen Anne school. This qualitative information was condensed into 19 statements about activities that a student had engaged in over the past 3 months, each requiring a binary “yes/no” response. Two statements were added that required a response on a 7-point rating scale (see the complete list of statements in the Supplementary Materials). Using the responses from 312 participants from the present sample we aimed to construct a potentially shorter self-report scale from a subset of the 19 statements that would fulfill the assumptions of the Rasch model. Testing for these three assumptions in an iterative fashion, we arrived at a subset of 8 items that met the Rasch model assumptions (exact version of the Martin–Loef test for item-homogeneity and unidimensionality: likelihood ratio = 7.02, *df* = 15, *p* = 0.96; Ponocny's test “T10” (Ponocny, 2001) for global subgroup invariance, *p* = 0.49; Ponocny's “T1” for local dependence assessing increased inter-item correlations indicated that there was no pair of items with a correlation of *p* < 0.05). The full set of items together with the endorsement frequencies of their binary response options and the selected set of eight items is given in the Supplementary Materials. Trait estimates for each participant were then extracted from the Rasch model and included in a principal component analysis together with the two rating scale items, all of which were highly correlated (values of Pearson's *r* between 0.52 and 0.75), with one component explaining 75% of the variance of the three variables. Component scores for each participant were then extracted from the principal component model and used as measures of the degree of concurrent musical activities of the participants in the study.

##### Academic and social self-concept

Academic self-concept and social self-concept were assessed with the subscales of the Multidimensional Self-Concept Scale (MSCS, Bracken et al., 2000) that are suitable for use with adolescents. Each subscale contains 20 statements with corresponding ratings scales. An age-appropriate normed score is derived from the aggregated ratings of each participant.

##### Academic self-theories and goals

The *Academic Self-Theories and Goals* questionnaire is produced by combining two short scales from Dweck (2000): the *Implicit Theories of Intelligence Scale for Children—Self Form* (short version) and the *Goal Choice* scale. Both scales have received experimental validation by a number of studies (e.g., Dweck et al., 1995; Dweck, 2000). As explained above, they measure the degree to which an individual believes that intelligence is fixed and unchangeable (*entity theory of intelligence*) or can be improved through hard work (*incremental theory of intelligence*). The Goals Choice scale measures the desire to succeed at tasks and demonstrate one's own ability (*performance goals*) and the desire to improve one's ability through taking on challenging tasks (*learning goals)*. Motivation to work hard at school derives significantly from both these types of goals in most individuals (Dweck, 2000). The questions from the two scales are interleaved and presented in the same order to all participants (see the Supplementary Materials for the complete question set). For six of the questions participants respond on a 6-point Likert; for the seventh question, the response option is binary. The two scales are scored independently, so each participant receives a *Theory of Intelligence* score and a *Goal Choice* score. High *Theory of Intelligence* scores (4–7) correspond to an entity theory of intelligence, while low scores (1–3) correspond to an incremental theory of intelligence; high *Goal Choice* scores (4–7) correspond to performance-goal orientation, whereas low *Goal Choice* scores (1–3) correspond to learning-goal orientation.

##### Musical self-theories and goals

The *Musical Self-Theories and Goals* questionnaire is the prototype for a new scale that assesses the participants' attitudes to the development of musical ability. Given that academic development is demonstrably influenced by students' theories of intelligence and their goal orientation (Dweck, 2000), the *Musical Self-Theories and Goals* questionnaire is constructed to assess two hypothetical analogous attitudes toward the development of musical ability. Firstly, the *Theory of Musical Ability* scale measures the belief that musical ability is fixed and unchangeable; this belief is termed an *entity theory of musical ability*. Alternatively, one might believe that musical ability can be significantly improved through hard work; this belief is termed an *incremental theory of musical ability*. Secondly, the *Musical Goals* scale measures the degree to which musical engagement is motivated by the two following goals: the desire to succeed at tasks and demonstrate one's own ability (*performance goals*) and the desire to improve one's ability through taking on challenging tasks (*learning goals*). Because the *Musical Self-Theories and Goals* questionnaire is intended to measure hypothetical constructs that are very similar to those measured by the *Academic Self-Theories and Goals* questionnaire, its questions are modeled closely on those of the latter, with academic terms being substituted for musical terms. However, this substitution process was not entirely trivial. In the case of assessing an individual's *theory of intelligence*, the term “intelligence” is widely understood by adults and young children as denoting an individual's cognitive abilities, but it is a term that bears little presumption about whether these cognitive abilities are innate or learned. Finding an analogous and well-understood term for the musical domain is more difficult. “Musical talent” is a well-understood term, but it implies a musical capacity that is innate (Boyle and Radocy, 1987). Likewise, “musical ability” is well-understood, but it implies an acquired musical capacity (Boyle and Radocy, 1987). The term “musical sophistication” (Ollen, 2006; Müllensiefen et al., 2014) is less loaded, but this term is not commonly used outside the scientific community. Similar problems apply to other terms frequently used in the academic literature such as “musical aptitude” (Karma, 2007), “musical gifts” Gagné (2003), “giftedness and talent” (McPherson and Williamon, 2006), “musical potential” (McPherson and Hallam, 2009) or “musicality” (Gembris, 1999). Instead of relying on one particular term to denote musical capacities, therefore, the *Musical Self-Theories and Goals* questionnaire makes use of a variety of idiomatic terms that ought to be well-understood by children and adults. It is proposed that using this variety of terms should smooth out some of the semantic loadings of the individual terms; this approach is argued to improve content validity at some small cost to internal reliability. The scale items can be found in the Supplementary Materials. Likewise, constructing a *musical goals* scale on the basis of the *Academic Goals* scale required a few changes to account for the different types of goals involved in musical and academic study. Unlike in the academic sphere, where performing well in exams is not necessarily the same as possessing a high academic ability, performing music well is arguably the definition of being a good musician. In the *Musical Self-Theories and Goals* questionnaire, therefore, the *Goal Choice* scale focuses less on musical performance itself and more on how the individual's musical abilities are recognized by others. The final scale can be found in the Supplementary Materials. Because not all participants in the present study were engaged in music education, some of the items in the *Musical Self-Theories and Goals* scale were not relevant to all of the participants, and so “Not Applicable” (NA) options were added to questions 1–6 of the questionnaire. The participants' instructions were: “Read each sentence below and select the one option that shows how much you agree with it. There are no right or wrong answers. If you feel that the question does not apply to you at all, you may select ‘Not Applicable.”’ Question 7 does not possess a “Not Applicable” option, since it' wording is intended to get the participant to commit to one particular choice, even if the two options are almost equally appealing. The *Musical Self-Theories and Goals* questionnaire is scored similarly to the *Academic Self-Theories and Goals* questionnaire: Each participant receives a *Theory of Musical Ability* score and a *Goal Choice* score. These scores are arrived at by reversing negative items and averaging individual item scores. Analogously to the *Academic Self-Theories and Goals* questionnaire, high *Theory of Musical Ability* scores (4–7) correspond to an entity theory of musical ability, while low scores (1–3) correspond to an incremental theory of musical ability; high *Goal Choice* scores (4–7) correspond to performance-goal orientation, whereas low *Goal Choice* scores (1–3) correspond to learning-goal orientation.

##### Academic performance

To measure academic performance we obtained end of year grades for all subjects that the students were taking during the school year. These grades ranged from 1 (lowest possible achievement) to 9 (highest possible). These grades were aggregated into five subject categories (Languages, Maths and Sciences, Social and Cultural, Aesthetic, Applied) following Gruber's (2009) classification scheme. We also obtained effort grades, reflecting the teacher's subjective assessment of the student effort invested into a particular subject and disregarding different ability levels; likewise, effort grades ranged from 1 (lowest possible effort) to 9 (highest possible).

For a list of all measures reported in this study see the first column of Table 1.

**Table 1:** **Bivariate matrix of Pearson correlation coefficients**.

	**Age**	**IQ**	**Melodic Memory**	**Beat Perception**	**Sound Similarity Perception**	**Concurrent Musical Activities**	**Musical Training**	**MusicalGoals**	**Theory of Musicality**	**AcademicGoals**	**Theory of Intelligence**	**Extraversion**	**Agreeableness**	**Conscientiousness**	**Emotional Stability**	**Openness**	**Academic Effort**	**Academic Achievement**	**Academic Self-Concept**
IQ	0.22^***^	1																	
MM	0.14^*^	0.3^***^	1																
BP	0.18^**^	0.08	0.29^***^	1															
SSP	−0.05	−0.07	−0.1	−0.01	1														
CMA	−0.08	0.14^*^	0.29^***^	0.18^**^	−0.05	1													
MT	−0.04	0.13^*^	0.35^***^	0.28^***^	0.01	0.75^***^	1												
MG	0.03	0.09	0.28^***^	0.14^*^	−0.08	0.35^***^	0.34^***^	1											
TM	0.05	0.07	0.25^***^	0.14^*^	−0.06	0.14^*^	0.09	0.35^***^	1										
AG	−0.04	0.11	0.08	0.05	−0.05	0.23^***^	0.18^***^	0.39^***^	0.18^**^	1									
TI	0.09	−0.03	0.11	0.1	−0.08	0.18^**^	0.07	0.28^***^	0.51^***^	0.3^***^	1								
EV	−0.29^***^	−0.18^**^	−0.2^***^	−0.08	0	0.03	0.02	−0.09	−0.03	−0.08	0.04	1							
AGR	−0.13^*^	−0.01	−0.2^*^	−0.04	0.02	0.07	0.03	0.09	0.06	0.19^***^	0.1	0.16^**^	1						
CSC	−0.06	0.02	0	0.1	0.08	0.13^*^	0.12^*^	0.13^*^	0.1	0.1	0.25^***^	0.14^*^	0.35^***^	1					
ES	−0.08	0.06	0	0.02	−0.04	0.02	0.05	0.1	0.11	0.19^***^	0.12^*^	0.32^***^	0.31^***^	0.27^***^	1				
O	−0.01	−0.02	−0.13^*^	0.05	−0.01	0.12^*^	0.13^*^	0.18^**^	0.12^*^	0.16^**^	0.15^**^	0.33^***^	0.34^***^	0.27^***^	0.24^***^	1			
AE	0.27^**^	0.22^*^	0.06	0.11	0	−0.06	0.1	−0.02	0.11	0.05	0.01	−0.05	0.14	0.43^***^	0.02	0.18	1		
AA	−0.1	0.26^***^	0.14^*^	0.19^**^	0.02	0.14^*^	0.15^*^	0.12	0.17^**^	0.12^*^	0.15^*^	0.03	0.07	0.32^***^	0.05	0.14^*^	0.8^***^	1	
ASC	−0.07	−0.26^***^	−0.2^**^	−0.18^**^	−0.09	−0.18^***^	−0.18^**^	−0.06	−0.07	−0.06	−0.05	0.15^**^	−0.1	−0.31^***^	−0.1	−0.06	−0.28^**^	−0.42^***^	1
SSC	−0.08	0.02	0.01	0.04	−0.07	0.02	0.1	0.12	0.15^**^	0.14^*^	0.15^**^	0.31^***^	0.15^**^	0.17^**^	0.34^***^	0.19^***^	0.2^*^	0.16^**^	0.2^***^

*Image specifications per type. Two-tailed significance levels are calculated on the basis of all available observations on a pair-wise basis and are not corrected for multiple comparisons. Significance levels are indicated as follows*:

* *p < 0.05*,

** *p < 0.01*,

*** *p < 0.001*.

*For the sake of an easier interpretation, the signs of the four self-theory scales (Musical Goals, Theory of Musicality, Academic Goals, Theory of Intelligence) have been reversed, where high values indicate strong beliefs in the incremental and changeable nature of musicality and intelligence as well as the importance of learning opportunities for setting musical and academic goals*.

## Results

As a first step we computed the pairwise correlation matrix between all 20 core variables of interest which is given in Table 1. Note that all 20 core variables had missing values. This was due to a number of reasons including a short temporary technical failure of the online testing platform, missing data on the school's marks database, and voluntary early termination of the testing session by a few participants. The number of missing values per variable varied from 5 to 45, except for academic effort, which had 210 missing values because academic effort is not collected consistently across all year groups at Queen Anne's school. The significance level of the bivariate correlations in Table 1 are computed on the basis of the pairwise complete observations for each variable pair.

Table 1 shows that 79 bivariate correlations are significant at the *p* < 0.05 level, with several being of a moderate size. However, the correlation matrix does not allow for much more insight and in particular it does not answer the question whether correlations between two variables would still be significantly different from zero once the influence from other correlated variables has been accounted for (i.e., once they are “partialled out”). Therefore, we made use of the PC algorithm (Spirtes et al., 2000; Pearl, 2009) as implemented in the R software package *pcalg* (Kalisch et al., 2012) to derive a graph that reflects the conditional independence relationships among the 20 variables in the dataset, implemented via partial correlation tests.

**Figure 1:**
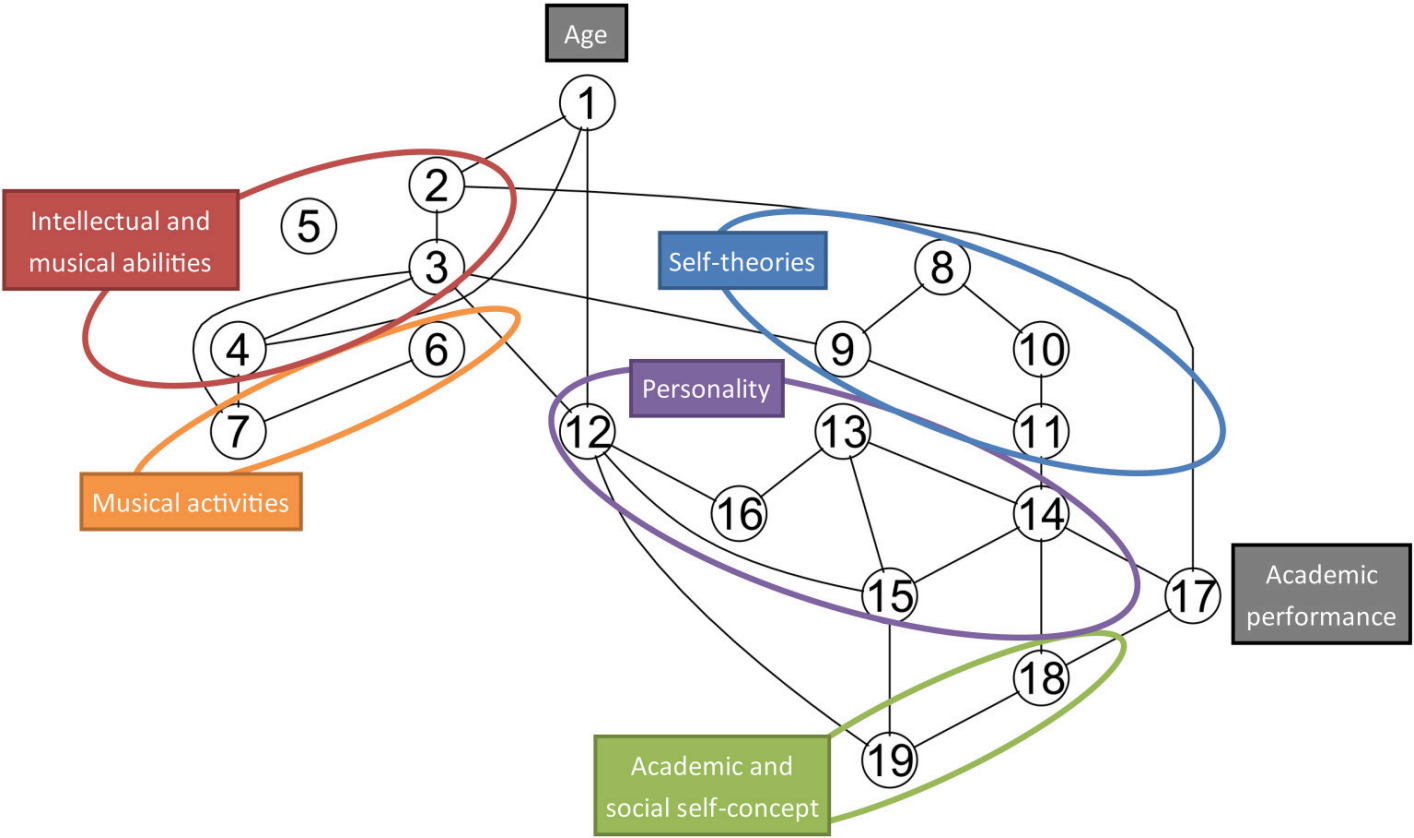
**Skeleton network of conditional independence relationship between the 19 core variables as derived from the PC algorithm (Spirtes et al., 2000; Kalisch et al., 2012)**. The variables are: 1, Age; 2, Intelligence; 3, Melodic Memory; 4, Beat Perception; 5, Sound Similarity Perception; 6, Concurrent Musical Activities; 7, Musical Training; 8, Musical Goals; 9, Theory of Musicality; 10, Academic Goals; 11, Theory of Intelligence; 12, Extraversion; 13, Agreeableness; 14, Conscientiousness; 15, Emotional Stability; 16, Openness; 17, Academic Achievement; 18, Academic Self-Concept; 19, Social Self-Concept.

We derived a skeleton graph form the PC algorithm that does not use any directed edges and therefore does not indicate directed causal relationships. However, the skeleton graph still indicates which variables are related to each other after taking into account the contribution from all other variables in partial correlation tests. A missing edge between two variables in the graph indicates that the two variables are not significantly related to each other conditional on the other variables in the network. Note that we excluded the variable Academic Effort from the network modeling because of its many missing values. Because of the differing numbers of missing values on the other 19 variables (i.e., between 307 and 262 observations per variable), we set the number of observations to *N* = 262 for the PC algorithm which represents a conservative lower bound. However, we also confirmed that choosing different values for N affected the resulting network only marginally. The network resulting from setting *N* = 307 contained 30 edges, while a network assuming only *N* = 262 had 28 edges, all of which were contained in the former network structure. An annotated version of the network graph with *N* = 262 is given in Figure 1.

The skeleton graph contains 28 edges which represent bivariate relationships that are significant at the *p* < 0.05 level after accounting for the influence of other variables via partial correlations. This contrasts with 79 correlations that are significant at the *p* < 0.05 level in the bivariate correlation matrix. This difference illustrates how a network arriving from partial correlations can suggest a very different picture compared to a pattern of bivariate correlations. The graph in Figure 1 contains several interesting relationships between pairs and groups of variables but due to space limitations we can only highlight seven of these relationships that are relevant to the hypotheses of this study:

(1) The four variables related to self-theories of musicality and intelligence (variables 8–11) form a closely connected cluster, supporting the hypotheses that these self-theories are conceptually related for the participants in our sample. Theory of intelligence (11) is indirectly related to academic achievement (17) through conscientiousness (14).

(2) As expected there are close connections between concurrent musical activities (6), the overall amount of musical training (7) and musical listening abilities [beat perception (4), melodic memory (3)]. There is an expected connection between performance on the melodic memory test (3) and theory of musicality (9). However, there is no direct connection between beat perception (4) and musical self-theories (8, 9).

(4) Intelligence (2) is related to melodic memory (3) and thus has a direct connection to musical listening abilities.

(5) Out of the five personality dimensions only extraversion (12) is directly related to a musical variable, namely beat perception ability (4). Openness to experience (16) is connected to the cluster of musical abilities and activities only indirectly via extraversion.

(6) Three variables are directly connected to academic achievement (17), including intelligence (2), conscientiousness (14) and academic self-concept (18).

(7) The graph contains a chain of connections from musical self-theories (8, 9) through theory of intelligence variables (10, 11), and conscientiousness (14) to academic achievement (17).

If we assume that the variables in this chain are causally linked then we can apply Pearl's do-calculus (Pearl, 1993, 2009) for estimating the effect of the two theory of musicality variables onto academic achievement (we used the function ida in the R package pcalg). Because from each of the two musical self-theory variables there are several possible routes to academic achievement as dependent variable, we obtain a multiset of effect sizes and the effect of theory of musicality on academic achievement is therefore described rather by a range of potential effect sizes which contains the true size. For the variable *Musical Goal Choice*, the effect size is estimated to be in the range of 0.07–0.12, meaning that for a difference of 1 standard deviation on the self-rating scale the academic achievement will change between 0.07 and 0.12 standard deviations. Similarly, for the variable *Theory of Musicality*, the effect size is estimated to lie between 0.07 and 0.15 standard deviations.

The effects of *Academic Self-Theories and Goals* variables on academic achievement lie in a similar range. For *Academic Goals* the effect size is between 0.09 and 0.12, while for *Theory of Intelligence* it is 0.07 and 0.15.

## Discussion

This study investigated how musical activities and abilities are related to intelligence, personality, self-concept and self-theories of musicality and intelligence as well as academic performance in a sample of female adolescents.

The main result of this study is a network model generated by the application of the PC algorithm to the correlational data. It does not provide any information about the directionality of causal effects but it demonstrates graphically which variables are directly or indirectly related to which other variables. As such the network model replicates some important findings form the existing literature. Among these are the association of intelligence with musical abilities (directly) and musical training (indirectly), which has been found in a number of previous studies (Schellenberg, 2006, 2011; Ruthsatz et al., 2008; Mosing et al., 2014b). The developmental connection between age and intelligence was also replicated in the network model (see Fry and Hale, 1996) and so did the association between intelligence and academic achievement that has been reported in previous studies (e.g., Laidra et al., 2007). Finally, concurrent musical activities, the degree of musical training and musical listening abilities (melodic memory and beat perception) are closely related which is in line with a vast number of studies showing a positive effect of musical training on musical listening abilities (see Müllensiefen et al., 2014 for results derived from a large sample in the general population).

In addition to replicating results from previous studies the network model also contains a number of novel results and findings that partly differ from previous studies. One example is the relationship between musical and personality variables. Many previous studies have found openness to experience to be the personality dimension that is most strongly (e.g., Vuoskoski and Eerola, 2011; Müllensiefen et al., 2014; Greenberg et al., 2015) related, or even the only variable significantly related to musical variables (Hunter and Schellenberg, 2011; Corrigall et al., 2013). However, in our results extraversion is the only Big 5 personality dimension directly connected to a musical variable and openness only has an indirect connection to musical variables via extraversion. Extraversion has been found to be correlated significantly with musical variables (see study 3 in Rentfrow and Gosling, 2006; Müllensiefen et al., 2014) and with preferences for certain musical genres (Rentfrow and Gosling, 2003) and styles of musical use and listening (Chamorro-Premuzic et al., 2009a,b). However, the result found in this study that extraversion separates musical variables from openness is novel. It is possible that this is a result of the female-only sample of this study, taking into consideration that substantial gender differences with regards to the association of personality and musical variables have been reported before (Werner et al., 2006). In addition, it is worth considering that personality is still malleable in adolescence (Vecchione et al., 2012) and that the associations with musical variables might change over the course of the teenage years. In this network model the important role of age is evidenced by the connection between age and extraversion. An interesting hypothesis implied in these findings concerns the causal direction of the relationship between music and openness. Instead of assuming openness as a stable trait to cause musical activity in adolescence (see Corrigall et al., 2013) it is conceivable that musical activities during the formative adolescent period in fact cause the development of openness to experience. This hypothesis would be in line with the analysis presented by Hille and Schupp (2015) who report significant effects of musical training during adolescence on openness as well as conscientiousness. While it is not possible to test the directionality hypothesis of the cause-effect relationship between music and personality with the current data, it constitutes a question that can potentially be answered from data to be collected in the envisaged longitudinal study.

Another novel finding arising from the network model are the relationships of the cognitive and musical self-theories. The four variables relating to self-theories of intelligence and musicality form a closely connected cluster, supporting the hypotheses that these self-theories are conceptually related for the participants in our sample. In addition, there is an expected connection between the entity/incremental self-theory of musicality performance on the melodic memory test. However, there is no direct connection between concurrent musical activities, musical training or beat perception ability and either of the musical self-theory variables. This seems to suggest that musical activities alone are not sufficient to influence or be influenced by musical self-theories, but that this relationship is mediated by at least one actual musical skill (melodic memory).

Furthermore, the graph shows that there is a chain of connections from musical self-theories through theory of intelligence and conscientiousness to academic achievement. An incremental theory of intelligence is indirectly associated with academic achievement through conscientiousness and effort. This finding confirms earlier work suggesting that an incremental theory of intelligence, that is, a belief that intelligence is malleable, is directly related to the level of conscientiousness and effort a student adopts and that this in turn relates to academic achievement (Diener and Dweck, 1980).

One of the aims of this present study was to consider what factors might contribute to the individual differences in student's motivation toward academic and musical studies. The analyses reveal that the beliefs about the nature of musicality and intelligence may relate to the level of conscientiousness. A similar finding is reported by Hille and Schupp (2015) who found a significant and positive effect of musical training during adolescence on conscientiousness, even after controlling for a large number of potentially confounding variables. In line with this, our network model suggests an interpretation where conscientiousness affects academic effort or motivation to work and that this in turn may have a bearing upon an adolescent's academic performance. Making the corresponding causal assumptions and applying Pearls' do-calculus to the network structure we demonstrate that both subscales of the theory of musicality had small to moderate effects on academic achievement. The effect sizes lie between 0.07 and 0.15 standard deviations, which is only slightly lower than the effects size of 0.18 standard deviations of musical training on average school grade reported by Hille and Schupp (2015) after controlling for confounding variables. Whether the causal assumptions are in fact justified and whether the effect of the self-beliefs about musicality are indeed in the order of about 0.1 standard deviations, can only be clarified through an interventional study which aims at deliberately changing self-beliefs and then observes potential changes in conscientiousness and ultimately academic performance.

One final outcome to mention is the network modeling methodology employed in this study, which makes use of the PC algorithm (Pearl, 1993, 2009) to identify a network of relationships that is consistent with the results of conditional independence tests, more specifically partial correlation tests. This approach is exploratory and related to the burgeoning field of causal structure discovery, which has produced very promising results in other areas (Park et al., 2006; Kalisch et al., 2010; Maathuis et al., 2010; Nagarajan et al., 2010; Wong et al., 2010). Highly multivariate datasets can generate models that are purely constructed from data and that represent a plausible middle ground between two other common forms statistical representation, large bivariate correlation matrices and regression models. The network model has the advantage that it is designed to handle correlations between variables in a principled way, thus illuminating relationships between potential confounders and the variables of main interest while avoiding strong *a priori* assumptions about the nature of dependent and independent variables in non-experimental datasets, as well as assumptions of homoscedasticity, and the absence of collinearity of regressors. Network models can be exploited even further to suggest the directions of causal effects if either certain conditions are met in the data (Pearl, 1993, 2009) or if convincing causal assumptions can be made (e.g., “age causes extraversion” but not the other way around). However, because of the limited sample size and the need to minimize the influence of sampling error on causal result, the perspective was not pursued in this study, but remains an option for the analysis of a larger sample of participants.

Finally, it is worth remembering that a severe limitation of this study is the fact the data was collected on a sample of female adolescents with a particular socio-economic background. It will be therefore necessary to extend the study on include male adolescents and students from other schools and social backgrounds in order to generalize results to the population of adolescents (at least in the UK). This is all the more important as gender effects with regards to musical training and instrument choice are well-documented in the literature (Hallam et al., 2008) and motivation to engage in musical activities appears to be closely connected to the development of gender related constructs (McClary, 1991).

Taken together, this study provides a baseline for the exploration of the inter-relatedness of self-theories of intelligence and musicality, personality traits, and ability in both academic and musical domains. Future work needs to extend this approach to a larger sample including different genders and different social backgrounds. In addition, the planned longitudinal study as well as any potential interventions that deliberately intend to change musical self-theories will seek to broaden our understanding of the nature and development of personality and musical abilities through adolescence and how these relate to academic motivation, effort and achievement. This seems like an ambitious endeavor at present, but has the potential to produce results that have important implications for the planning of academic curricula, the functional use of music education and the role that music plays in the development of personality and cognitive abilities in the formative period of adolescence.

### Conflict of interest statement

The authors declare that the research was conducted in the absence of any commercial or financial relationships that could be construed as a potential conflict of interest.
