# Supplementary material for: Investigating the importance of self-theories of intelligence and musicality for students' academic and musical achievement
Source: Front Psychol. 2015 Nov 5;6:1702. doi: 10.3389/fpsyg.2015.01702 (PMC4633492; doi:10.3389/fpsyg.2015.01702)
Supplement: Supplementary file 1 [file DataSheet1.DOCX]

Supplementary Material

Investigating the importance of self-theories for students’ academic and musical development

Daniel Müllensiefen1*, Peter Harrison1, Francesco Caprini1, Amy Fancourt2

^1^Department of Psychology, Goldsmiths, University of London, United Kingdom

^2^Head of Psychology, Queen Anne’s School, Reading

***Correspondence:** Daniel Müllensiefen, Department of Psychology, Goldsmiths, University of London, New Cross Road, London, SE8 4AW, United Kingdom.

d.mullensiefen@gold.ac.uk

# Ten Item Personality Inventory (TIPI, Gosling, Rentfrow & Swann, 2003) – extended

1. extraverted, enthusiastic + *sociable, lively*

2. critical, quarrelsome + *grumpy, selfish*

3. dependable, self-disciplined + *responsible, persistent*

4. anxious, easily upset + *touchy, fearful*

5. open to new experiences, complex + *curious, thoughtful*

6. reserved, quiet + *shy, private*

7. sympathetic, warm + *kind, patient*

8. disorganised, careless + *lazy, irresponsible*

9. calm, emotionally stable + *independent, peaceful*

10. conventional, uncreative + *shallow, simple*

# Concurrent Musical Activities

For answering the following questions below, please only think about the musical activities you did within the last three months.

At the moment, do you   ... [No / Yes ]

**Table 1. Concurrent Musical Activities**

| **Musical Activity** | **No (%)** | **Yes (%)** |
| --- | --- | --- |
| sing in a choir | 0.6389776 | 0.3610224 |
| play in a band | 0.91693291 | 0.08306709 |
| *play in an orchestra | 0.9201278 | 0.0798722 |
| play in a small ensemble | 0.8338658 | 0.1661342 |
| produce music on the computer | 0.6900958 | 0.3099042 |
| *sometimes play music with a friend | 0.4185304 | 0.5814696 |
| sometimes compose or arrange music | 0.7220447 | 0.2779553 |
| *sometimes make music at events or special occasions | 0.8434505 | 0.1565495 |
| *receive individual lessons on an instrument (or voice) | 0.514377 | 0.485623 |
| *receive group lessons on an instrument (or voice) | 0.8945687 | 0.1054313 |
| teach yourself to play an instrument (or sing) | 0.6741214 | 0.3258786 |
| *have music classes in school | 0.4313099 | 0.5686901 |
| attend extracurricular music theory/appreciation classes | 0.8913738 | 0.1086262 |
| *attend music-related after school clubs | 0.798722 | 0.201278 |
| *sometimes compile playlists for myself or others | 0.543131 | 0.456869 |
| follow other people’s playlists | 0.5654952 | 0.4345048 |
| sometimes act as DJ at parties | 0.8913738 | 0.1086262 |
| follow music shows on radio, TV or internet | 0.4345048 | 0.5654952 |
| swap music files with other people | 0.7955272 | 0.2044728 |
| None of the above | 0.96805112 | 0.03194888 |

Note. The 8 items finally selected for the model and the scoring are marked with *.

**Two added statements:**

1. How much do you currently practice your instrument(s)? 0 / 0-15 / 15-30 / 30-60 / 60-90 / 90-120 / more than 120 minutes per day
 
2. Taken all together (own practice, rehearsals, instrument lessons, gigs etc.) how much time do you spend making music per week at the moment? 0 / 0-1 / 1-2  / 2-4 / 4-7 / 7-14 / more than 14 hours per week

# Self- Theories and Goals

# Table 2. *The* Academic Self-Theories and Goals *Questionnaire*

| No. | Question | Scale | Response options | Scoring |
| --- | --- | --- | --- | --- |
| 1 | You have a certain amount of intelligence, and you really can't do much to change it. | *Theory of Intelligence* | 6-level Likert item, agreement/ disagreement | Positive |
| 2 | If I knew I wasn't going to do well at a task, I probably wouldn't do it even if I might learn a lot from it. | *Academic Goals* | 6-level Likert item, agreement/ disagreement | Positive |
| 3 | Your intelligence is something about you that you can't change very much. | *Theory of Intelligence* | 6-level Likert item, agreement/ disagreement | Positive |
| 4 | Although I hate to admit it, I sometimes would rather do well in a class than learn a lot. | *Academic Goals* | 6-level Likert item, agreement/ disagreement | Positive |
| 5 | It's much more important for me to learn things in my class than it is to get the best grades. | *Academic Goals* | 6-level Likert item, agreement/ disagreement | Negative |
| 6 | You can learn new things, but you can't really change your basic intelligence. | *Theory of Intelligence* | 6-level Likert item, agreement/ disagreement | Positive |
| 7 | If I had to choose between getting a good grade and being challenged in class, I would choose... | *Academic Goals* | Binary choice |  |

**Table 3*. The* Musical Self-Theories and Goals *Questionnaire***

| No. | Question | Scale | Response options | Scoring |
| --- | --- | --- | --- | --- |
| 1 | You have a certain amount of musicality and you really can't do much to change it. | *Theory of Musical Ability* | 6-level Likert item, agreement/ disagreement; NA option | Positive |
| 2 | If I knew I wasn't going to do well at a musical activity, I probably wouldn't do it even if I might learn a lot from it. | *Goal Choice* | 6-level Likert item, agreement/ disagreement; NA option | Positive |
| 3 | People are born with very different amounts of musical talent, and practice does little to change that. | *Theory of Musical Ability* | 6-level Likert item, agreement/ disagreement; NA option | Positive |
| 4 | Although I hate to admit it, I sometimes would rather do well in music exams than get better at music itself. | *Goal Choice* | 6-level Likert item, agreement/ disagreement; NA option | Positive |
| 5 | It's much more important for me to develop my musical skills than to get recognised for my musical skills. | *Goal Choice* | 6-level Likert item, agreement/ disagreement; NA option | Negative |
| 6 | Musical success depends more on your innate talents than on how much practice you do. | *Theory of Musical Ability* | 6-level Likert item, agreement/ disagreement; NA option | Positive |
| 7 | If I had to choose between performing easy music that I know I can do well or performing music that challenges me, I would choose… | *Goal Choice* | Binary choice; no NA option | see Table 4 |

**Table 4. *Response Options for Question* 7 *of the* Musical Self-Theories and Goals *Questionnaire***

| Score | Label |
| --- | --- |
| 4 | “easy music” |
| 3 | “challenging music” |
